# The Association of Methylenetetrahydrofolate Reductase Genotypes with the Risk of Childhood Leukemia in Taiwan

**DOI:** 10.1371/journal.pone.0119776

**Published:** 2015-03-20

**Authors:** Jen-Sheng Pei, Chin-Mu Hsu, Chia-Wen Tsai, Wen-Shin Chang, Hong-Xue Ji, Chieh-Lun Hsiao, Chia-En Miao, Yuan-Nian Hsu, Da-Tian Bau

**Affiliations:** 1 Department of Pediatrics, Taoyuan General Hospital, Ministry of Health and Welfare, Taoyuan, Taiwan, ROC; 2 Terry Fox Cancer Research Laboratory, China Medical University Hospital, Taichung, Taiwan, ROC; 3 Graduate Institute of Basic Medical Science, China Medical University, Taichung, Taiwan, ROC; 4 Graduate Institute of Clinical Medical Science, China Medical University, Taichung, Taiwan, ROC; 5 Department of Family Medicine, Taoyuan General Hospital, Ministry of Health and Welfare, Taoyuan, Taiwan, ROC; The Institute of Cancer Research, UNITED KINGDOM

## Abstract

**Background:**

Acute lymphoblastic leukemia (ALL) is the most prevalent type of pediatric cancer, the causes of which are likely to involve an interaction between genetic and environmental factors. To evaluate the effects of the genotypic polymorphisms in methylenetetrahydrofolate reductase (*MTHFR*) on childhood ALL risk in Taiwan, two well-known polymorphic genotypes of *MTHFR*, C677T (rs1801133) and A1298C (rs1801131), were analyzed to examine the extent of their associations with childhood ALL susceptibility and to discuss the *MTHFR* genotypic contribution to childhood ALL risk among different populations.

**Methodology/Principal Findings:**

In total, 266 patients with childhood ALL and an equal number of non-cancer controls recruited were genotyped utilizing PCR-RFLP methodology. The *MTHFR* C677T genotype, but not the A1298C, was differently distributed between childhood ALL and control groups. The CT and TT of *MTHFR* C677T genotypes were significantly more frequently found in controls than in childhood ALL patients (odds ratios=0.60 and 0.48, 95% confidence intervals=0.42–0.87 and 0.24–0.97, respectively). As for gender, the boys carrying the *MTHFR* C677T CT or TT genotype conferred a lower odds ratio of 0.51 (95% confidence interval=0.32–0.81, P=0.0113) for childhood ALL. As for age, those equal to or greater than 3.5 years of age at onset of disease carrying the *MTHFR* C677T CT or TT genotype were of lower risk (odds ratio= 0.43 and 95% confidence interval=0.26–0.71, P=0.0016).

**Conclusions:**

Our results indicated that the *MTHFR* C677T T allele was a protective biomarker for childhood ALL in Taiwan, and the association was more significant in male patients and in patients 3.5 years of age or older at onset of disease.

## Introduction

Acute lymphoblastic leukemia (ALL) is now the most common form of pediatric leukemia, accounting for 25–30% of all childhood malignancies [1]. The annual incidence rate worldwide of childhood ALL is approximately 10 cases per 100,000, with peak incidence occurring at approximately 2 to 5 years of age [2]. While the clinical, pathological and immunophenotypic features of the disease are well documented, the etiology of ALL has not been fully clarified [1]. In the literature, certain environmental factors (i.e., ionizing radiation, parental use of alcohol and tobacco, and virus exposure) have been identified as potential risk factors for the development of childhood ALL, but only ionizing radiation has been confirmed thus far [3]. However, several lines of evidence now suggest that genetic factors may play a significant role in the development of childhood ALL. For instance, inherited genetic disorders, such as Down syndrome and Fanconi anemia, have been associated with an enhanced ALL risk [4, 5]. Additionally, genetic mutations in several cancer-related genes, such as *p53*, *N-ras*, and *PHF6*, have frequently been identified in ALL patients [6]; and finally, only a small fraction of children who are exposed to environmental factors go on to develop ALL, indicating the potential for a genetic predisposition to develop childhood ALL.

To reveal the environmental and genomic factors together with the interactions among them is useful for evaluation and prevention of cancer risk. It is believed that one group of the candidate genes are those encoding enzymes related to the metabolism of identified carcinogens. Among these genes, methylenetetrahydrofolate reductase (MTHFR) is a folate-metabolism enzyme in charge of the conversion of 5, 10-methylene-tetrahydrofolate into 5-methyltetrahydrofolate, homocysteine remethylation, and biosynthesis of DNA and RNA [7]. The regulatory effects of MTHFR on DNA methylation, DNA replication, DNA repair and cell division make *MTHFR* a potential candidate for a cancer-predisposition gene. It is reasonable that rapidly proliferating malignancies have a higher requirement for DNA synthesis and could be more susceptible to folate deficiency and resultant DNA damage. Low dietary folate and MTHFR deficiency induced the formation of intestinal tumors in a BALB/c mice model [8].

Previous investigations of *MTHFR* variations focused on the catalytic domain and the two polymorphisms, C677T (rs1801133) and A1298C (rs1801131), which may determine its enzymatic activity [9, 10]. In the case of the C677T polymorphism, the cytosine base at position 677 changes to a thymidine base, which in turn affects the amino acid at position 222, as it is changed from alanine to valine. The MTHFR enzyme TT variants from the polymorphism become thermo labile, resulting in a loss of its activity with elevated temperature [11]. The modified protein loses its flavin adenine dinucleotide cofactor more quickly and has a lower stability. The mutation effect can be suppressed by the addition of folate, which causes a higher flavin adenine dinucleotide affinity and an increase in MTHFR stability [11]. The *MTHFR* A1298C polymorphism is localized in the coding regulatory domain [12]. In the literature, it was reported that heterozygotes and rare homozygotes of MTHFR C677T variant exert 60% and 30% of wild-type enzyme activity, respectively [10]. As for A1298C, the rare homozygous have 60% of wild-type activity [10]. In 2004, it was found that cancer cells which expressed 677T MTHFR were of lower activity than those express 677C MTHFR. Also the expression of mutant MTHFR 677T would increase the sensitivity of cancer cells to the cytotoxicity of 5FU. In null mice model, the expression of 677T MTHFR enhanced the growth rates of xenografts than those expressed wild-type 677C MTHFR. Consistent with the evidence observed in cell models, the 677T xenografts were more sensitive to 5FU treatment than those of 677C in mice model [13]. Many studies investigating the *MTHFR* variant have found positive associations with solid cancers, such as colorectal cancer [14], breast cancer [15], oral cancer [16, 17], and lung cancer [18].

Over the past decade, there has been a growing interest in the possible association between folate-related polymorphisms and the risk of developing lymphoid malignancies, including childhood ALL. First, Skibola [19] and Matsuo [20] in 1999 and 2001, respectively, reported that people with variant *MTHFR* genotypes had a significantly lower susceptibility to adult ALL and malignant lymphoma. In the same period, Franco [21] and Wiemels [22] provided similar pilot results for pediatric leukemia. The purpose of this study, therefore, was to analyze the genetic polymorphisms of both *MTHFR* C677T and A1298C in a representative pediatric population sample (control/case = 266/266), to investigate the correlation between *MTHFR* genotypes and childhood ALL in Taiwanese children, and to then summarize all of the relevant updated literature.

## Materials and Methods

### Study population and sample collection

Our study was approved by the Institutional Review Board of China Medical University Hospital, and written informed consent was obtained from one or both the parent of all participants. Two hundred and sixty-six patients diagnosed with childhood ALL (all patients under 18 years of age) were recruited between Apr 2005 to Jan 2010 from the general surgery outpatient clinics within the Pediatric Departments at China Medical University Hospital and National Taiwan University Hospital, Taiwan, Republic of China. All of the clinical characteristics of these ALL patients, including their histological details, were identified by expert surgeons. All subjects voluntarily participated, completed a questionnaire with the help of parents or guardians and provided peripheral blood samples. The questionnaires recorded their disease history, diet and sleep lifestyles and the disease history, diet and behavioral lifestyle, social-economic status of the parents. An equal number of age-matched non-cancer healthy volunteers were selected for use as a control group following initial random sampling from the Health Examination Cohort established from Apr 2005 to Jan 2010 as previously published [23]. The registered health practitioners in the hospital provide a multidisciplinary team approach of health assessment for the volunteers. Most of the volunteers underwent health examinations every 5 to 6 months. A total of 457 volunteers age under 18 years were recruited into this study and chosen were cancer free by the age at diagnosis of the case child with the International Classification of Disease, ninth revision (ICD-9) codes. Finally, 266 participants were included for analysis in the study since we have to match the population structure (number, age and gender) with our case population. The overall agreement rate in the study was above 85%.

### Genotyping assays

Genomic DNA was prepared from peripheral blood leukocytes using a QIAamp Blood Mini Kit (Blossom, Taipei, Taiwan), long-term stored at -80°C, diluted and aliquotted for genotyping as working stock at -20°C [23–25]. Genotyping for *MTHFR* C677T and A1298C of all subjects was carried out by polymerase chain reaction restriction fragment length polymorphism (PCR-RFLP) assays as previously published [17, 25–27]. The primers for *MTHFR* C677T were forward 5’- TGA AGG AGA AGG TGT CTG CGG GA-3’ and reverse 5’- AGG ACG GTG CGG TGA GAG TG-3’. The primers for *MTHFR* A1298C were forward 5’- GGG AGG AGC TGA CCA GTG CAG-3’ and reverse 5’- GGG GTC AGG CCA GGG GCA G-3’. The underlined C mismatched base in the forward primer were used to create a partial *Fnu4H I* cutting site, which will be completed in the presence of the C allele at the A1298C polymorphic site. The following cycling conditions were performed: 5 min of initial denaturation at 95°C, 35 cycles of 30 sec of denaturation at 95°C, 30 sec of annealing at 54°C and 1 min of elongation at 72°C, and 7 min of final extension at 72°C. The 198-bp PCR product of *MTHFR* C677T and 138-bp PCR product of *MTHFR* A1298C were subject to enzyme digestion with *Hinf I* and *Fnu4H I* (New England, Biolabs, Beverly, MA USA), respectively for 4 h and then visualized by ethidium bromide-stained 3% agarose gel electrophoresis under UV light. On digestion with *Hinf I*, the PCR product of *MTHFR* C677T arising from the C allele was uncut (198 bp), whereas the T allele was cut into fragments of 175 bp and 23 bp. On digestion with *Fnu4H I*, the PCR product of *MTHFR* A1298C arising from the A allele was uncut (138 bp), whereas the C allele was cut into fragments of 119 bp and 19 bp [17, 25, 27]. The success rate of PCR-RFLP is 100%, and the genotypes of five percent of the participants in both the control and patient groups were analyzed by PCR direct sequencing (Genomics BioSci & Tech Co., Taipei). The consistency between direct sequencing and PCR-RFLP was 100%.

### Statistical analyses

Only those participants having both genotypic and clinical data (control/case = 266/266) were selected for final analysis. The descriptive statistics of patients and controls were presented as the mean and standard deviations (SDs) or as percentages. The Pearson’s chi-square test or Fisher’s exact test (when any cell was less than five) was used to compare the distribution of the genotypes. Associations were expressed and evaluated as odds ratios (ORs) with 95% confidence intervals (95%CIs). Interactions between the genotypes and onset age or gender were examined by using the likelihood ratio test. Statistical tests were deemed significant when the *P*-value was less than 0.05. All statistical analysis were performed with SAS 9.2 and SPSS 17.

## Results

The frequency distributions for the age and gender of 266 childhood ALL patients and 266 non-cancer controls are shown in Table 1. The characteristics of the patients and controls were well matched (*P*>0.05) (Table 1).

**Table 1:** Demographic data of 266 childhood ALL patients and 266 controls.

Characteristic	Controls (n = 266)			Patients (n = 266)			*p-value* ^a^
	n	%	Mean (SD)	n	%	Mean (SD)	
Age (years)			8.3 (4.8)			7.0 (4.4)	0.64
Gender							1.00
Boy	148	55.6%		148	55.6%		
Girl	118	44.4%		118	44.4%		

^a^ Based on a chi-square test.

The genotype frequencies for the *MTHFR* C677T and A1298C in the controls and childhood ALL patients are shown in Table 2. The genotype frequencies of the two *MTHFR* SNPs of the controls were in Hardy-Weinberg Equilibrium (*p* = 0.9007 and 0.8886, respectively). The genotypic frequency distributions for *MTHFR* C677T were significantly different between childhood ALL and control groups (*P* = 0.0076), while those for the A1298C polymorphism were not significantly different (*P*>0.05) (Table 2). Those who carried CT, TT, CT or TT genotypes had significantly reduced risk of ALL with ORs of 0.60, 0.48, and 0.58 respectively compared to those with the CC genotype (95% CI = 0.42–0.87, 0.24–0.97 and 0.41–0.82, respectively). The conclusion that can be deduced from Table 2 is that the *MTHFR* C677T T allele seems to be a protective biomarker for childhood ALL in Taiwan.

**Table 2:** Distribution of the *MTHFR* genotypes among 266 childhood ALL patients and 266 controls.

Genotype	Controls	%	Cases	%	*P-*value^a^	OR (95% CI)^b^
C677T rs1801133					0.0076*	
CC	134	50.4%	169	63.5%		1.00 (Reference)
CT	109	41.0%	83	31.2%		0.60 (0.42–0.87)*
TT	23	8.6%	14	5.3%		0.48 (0.24–0.97) *
CT+TT	132	49.6%	97	36.5%		0.58 (0.41–0.82) *
A1298C rs1801131					0.8984	
AA	171	64.3%	168	63.2%		1.00 (Reference)
AC	85	32.0%	86	32.3%		1.03 (0.71–1.49)
CC	10	3.7%	12	4.5%		1.22 (0.51–2.90)
AC+CC	95	35.7%	98	36.8%		1.05 (0.74–1.50)

^a^ Based on Pearson’s chi-square test

^b^ OR: odds ratio; CI: confidence interval

* Statistically significant

Because age and gender are the predominant risk factors for developing childhood ALL, the interactions between the *MTHFR* genotype and age and gender were further analyzed and presented in Table 3. The average age of onset for the 133^rd^ and 134^th^ subjects in the control and patient groups was 3.5 years; thus, we further stratified the groups into <3.5 and ≥3.5 year-old subgroups. Noticeably, in the elder (≥ 3.5 years) group, subjects with CT or TT genotypes for *MTHFR* C677T had lower risks for developing childhood ALL than those with the homozygous CC genotype (*P* for trend = 0.0016, OR = 0.48 and 0.22, CI = 0.28–0.80 and 0.07–0.69 for CT and TT, respectively); however, this was not the case for the younger (<3.5 years) group (Table 3). As for gender, boys with CT or TT genotypes for *MTHFR* C677T were less likely to develop childhood ALL than those with the homozygous CC genotype (*P* for trend = 0.0113, OR = 0.54 and 0.32, CI = 0.33–0.89 and 0.11–0.94 for CT and TT, respectively), but this was not the case for the girls (Table 3). In summary, analyses revealed an interaction between the age of onset and gender among *MTHFR* C677T genotypes in the childhood ALL susceptibility (*P* values for interaction = 0.0378 and 0.2524 for ≥3.5 years versus < 3.5 years and girls versus boys, respectively).

**Table 3:** Distribution of the *MTHFR* C677T and A1298C genotypes stratified by age and gender

	*MTHFR* C677T						*MTHFR* A1298C				
Characteristics	Controls	Cases	*P*_*trend*_ ^a^	*P*_*inter*_ ^b^	OR (95% CI)^c^	Characteristics	Controls	Cases	*P*_*trend*_ ^a^	*P*_*inter*_ ^b^	OR (95% CI) ^c^
	n (%)	n (%)					n (%)	n (%)			
**Onset age**						**Onset age**					
< 3.5 years			0.5812	**0.0378**		< 3.5 years			0.8519	NS	
CC	70 (52.63)	78 (58.65)			1.00 (Reference)	AA	83 (62.41)	86 (64.66)			1.00 (Reference)
CT	53 (39.85)	45 (33.83)			0.76 (0.46–1.27)	AC	44 (33.08)	40 (30.08)			0.88 (0.52–1.48)
TT	10 (7.52)	10 (7.52)			0.90 (0.35–2.28)	CC	6 (4.51)	7 (5.26)			1.13 (0.36–3.49)
CT+TT	63 (47.37)	55 (41.35)			0.78 (0.48–1.27)	AC+CC	50 (37.59)	47 (35.34)			0.91 (0.55–1.50)
≥3.5 years			0.0016*			≥3.5 years			0.7370		
CC	64 (48.12)	91 (68.42)			1.00 (Reference)	AA	88 (66.16)	82 (61.65)			1.00 (Reference)
CT	56 (42.11)	38 (28.57)			0.48 (0.28–0.80)*	AC	41 (30.83)	46 (34.59)			1.20 (0.72–2.02)
TT	13 (9.77)	4 (3.01)			0.22 (0.07–0.69)*	CC	4 (3.01)	5 (3.76)			1.34 (0.35–5.17)
CT+TT	69 (51.88)	42 (31.58)			0.43 (0.26–0.71)*	AC+CC	45 (33.84)	51 (38.35)			1.22 (0.74–2.01)
**Gender**						**Gender**					
boys			0.0113*	NS		boys			0.7082	NS	
CC	76 (51.35)	100 (67.57)			1.00 (Reference)	AA	96 (64.86)	90 (60.81)			1.00 (Reference)
CT	60 (40.54)	43 (29.05)			0.54 (0.33–0.89)*	AC	47 (31.76)	51 (34.46)			1.16 (0.71–1.89)
TT	12 (8.11)	5 (3.38)			0.32 (0.11–0.94)*	CC	5 (3.38)	7 (4.73)			1.49 (0.46–4.88)
CT+TT	72 (48.65)	48 (32.43)			0.51 (0.32–0.81)*	AC+CC	52 (35.14)	58 (39.19)			1.19 (0.74–1.91)
girls			0.3565			girls			0.9130		
CC	58 (49.15)	69 (58.47)			1.00 (Reference)	AA	75 (63.56)	78 (66.10)			1.00 (Reference)
CT	49 (41.53)	40 (33.90)			0.69 (0.40–1.18)	AC	38 (32.20)	35 (29.66)			0.89 (0.51–1.55)
TT	11 (9.32)	9 (7.63)			0.69 (0.27–1.77)	CC	5 (4.24)	5 (4.24)			0.96 (0.27–3.46)
CT+TT	60 (50.85)	49 (41.53)			0.69 (0.41–1.15)	AC+CC	43 (36.44)	40 (33.90)			0.89 (0.52–1.53)

^a^ *P* for trend based on chi-square test.

^b^ *P* for interaction based on likelihood ratio test; NS, non-significant.

^c^ OR, odds ratio; CI, confidence interval.

* Statistically significant.

## Discussion

ALL is more common among children than adults. Among the patients, the precursor B subtype accounted for approximately 75% to 90% of the cases, while the remaining 10% to 25% were precursor T lymphoblastic leukemia [2]. Lymphocytic leukemia cells are rapidly dividing cells that require higher folate supplementation and are more vulnerable to folate deficiency. Previous research has demonstrated that maternal folate supplementation during pregnancy can be effective in reducing the risk of childhood ALL in babies [28]. The two most common SNPs of the *MTHFR* gene may determine MTHFR enzyme activity and the availability of folate to the whole body and may ultimately be linked to childhood ALL risk. For this reason, we investigated their relationships with the susceptibility to developing childhood ALL in Taiwan. We found that the T variant genotypes of *MTHFR* C677T were significantly associated with a lower susceptibility to childhood ALL (Table 2). These findings are consistent with previous research that identified the T allele to be a protective factor [21, 22, 29–36], but not with those studies which identified the T allele as a risk factor [37–39]. Additionally in some studies the T allele has been shown to have no association with childhood ALL [40–54]. Table 4 contains a summary of the findings from all of the literature investigating the association between *MTHFR* genotypes and childhood leukemia risk with a representative sample size (control/case larger than 70/70) and a non-redundant population. Among the studies, allele T was associated with decreased risk in populations of Taiwan, Serbia, China, Netherlands, Greece, Canada, and UK [22, 30–32, 34, 36], while with increased risk in populations of India [38]. Others proposed that the T allele was not associated with childhood ALL [40–54]. However, even the people in the same country, for instance Brazil, there was a dramatically different susceptibility to cancer, showing both decreased [21, 35] and increased risk [37]. In India, the same inconsistency could be observed [38, 39, 42, 45]. This kind of inconsistency may be mainly caused by different genetic background and environmental exposure status in addition to sampling bias. Therefore, all the molecular epidemiologists are revealing the genomic and environmental factors contribute to childhood ALL. The limited sample sizes in childhood ALL, compared to studies using larger amounts of samples collected among patients of common cancers, may be one of the factors contributing to inconsistent conclusions. For the low incidence of childhood ALL, this is unavoidable. In a current meta-analysis investigating the contributions of *MTHFR* C677T and A1298C genotypes to childhood ALL, it was demonstrated that the T allele of *MTHFR* C677T was associated with a lower risk of childhood ALL in both Asians and Caucasians, while A1298C was not observed to have any effect [55]. The current study was the first to utilize a Taiwanese sample, and the relationship was consistently positive for *MTHFR* C677T and negative for A1298C (Table 2).

**Table 4:** Summary of the original international literature investigating the association of the *MTHFR* C677T genotypes with childhood leukemia.

First author	Ref #	Year	Population	Controls (n)	Cases (n)	Association and highlights
Pei	Current study	2014	Taiwan	266	266	Allele T associated with lower risk, especially in boys gender and the age population who were equal to or elder than 3.5 years old
Li	54	2014	China	93	98	No association
Silva	37	2013	Brazil	390	177	Allele T associated with higher risk
Amigou	40	2012	France	1681	764	No association
Azhar	41	2012	Iran	109	72	No association
Nikbakht	42	2012	India	100	125	No association
Chan	29	2011	Indonesia	177	185	Specific haplotypes of MTHFR C677T and A1298C (C-C & T-A) associated with a reduced risk
Metayer	43	2011	35 countries	448	377	No association
Damnjanovic	30	2010	Serbia	412	78	Allele T associated with lower risk
Lightfoot	44	2010	UK	824	939	No association
Sadananda	45	2010	India	99	86	No association
Sood	38	2010	India	255	95	Allele T associated with higher risk
Tong	31	2010	China	508	361	Allele T associated with lower risk
Yeoh	46	2010	Chinese/Malay	756	531	No association
de Jonge	32	2009	Netherlands	496	245	Allele T associated with lower risk
Alcasabas	47	2008	Philippines	394	189	No association
Kamel	33	2007	Egypt	311	88	Specific haplotypes of MTHFR C677T and A1298C (677CT and 1298AC) associated with a reduced risk
Petra	48	2007	Slovenia	258	68	No association
Kim	49	2006	Korea	100	66	No association
Chatzidakis	34	2006	Greece	88	52	Allele T associated with lower risk
Reddy	39	2006	India	142	135	Allele C associated with lower risk, and male children more susceptible to ALL
Zanrosso	35	2006	Brazil	199	176	Allele T associated with lower risk
Oliveira	50	2005	Portugal	111	103	No association
Schnakenberg	51	2005	Germany	379	443	No association
Thirumaran	52	2005	Germany	1472	460	No association
Krajinovic	36	2004	Canada	330	270	Allele T associated with lower risk
Wiemels	22	2001	UK	200	253	Allele T associated with lower risk
Franco	21	2001	Brazil	71	71	Allele T associated with lower risk

Note: Some studies that had less than 70 cases and 70 controls of a redundant population were not included.

The survey of literature was updated 2014/09/18.

We have further analyzed the relationship between the C677T genotype and childhood ALL risk according to the subject age and gender. Interestingly, the interaction between *MTHFR* C677T and age is clear; specifically children ≥3.5 years old at age of onset of disease with a CT or TT genotype had a lower risk of childhood ALL than those with the CC genotype. This relationship was not found for the group of children <3.5 years old at age of onset of disease (Table 3). Additionally, no such age difference was observed in analyses of the A1298C genotype. In 2013, Jiang and his colleagues extended the Meta-analysis to 37 individual studies investigating adult ALL. They found that TT genotype was associated with a lower risk of ALL (OR = 0.776, 95% CI: 0.687–0.877, *p*< 0.001). After stratification by ethnicity, the significance only existed among Caucasians (OR = 0.715, 95% CI: 0.655–0.781, *p*< 0.001), at borderline among Asian (OR = 0.711, 95% CI: 0.591–1.005, *p* = 0.055), but not among others (OR = 0.913, 95% CI: 0.656–1.271, *p* = 0.590) [56]. The mechanisms of MTHFR involved in the etiopathology of and progression of ALL may be different for adults and children. Although no statistically significance was found in the girls-only analysis in Table 3, there is a similar trend to that observed for the boys (Table 3). The enlarged sample size may provide more realistic answer. Thus, as for the different contribution of *MTHFR* genotypes to ALL susceptibility between boys and girls, further investigations are in urgent need. In the literature, studies have revealed significant differences in the serum or plasma folate levels among people with various C677T genotypes, but not among those with various A1298C genotypes [57, 58]. Further measurement and analysis of the folate levels among children of different ages, together with their diet intake of folic acid, may help us to better understand the etiology.

We also tested for a gender-dependent effect on determining childhood susceptibility to ALL. The protective impact of the T allele at MTHFR C667T with respect to cancer risk appeared to be stronger for boys than the girls (Table 3) but this difference was not statistically significant. The incidence of pediatric hematological malignancies worldwide has increased for boys but not for girls [59, 60]. While the complete underlying mechanism has not been discovered, sex hormones and steroids reportedly play a part in the control of the proliferation of leukemic cells. Supporting the idea of gender differences in susceptibility to childhood ALL, it was found that 17-βestrogen had a stronger inhibitory effect than testosterone on human monoblastic U937 cells [61]. In addition, the TT genotype at MTHFR C677T conferred higher plasma homocysteine levels than the CC genotype selectively in folate-dependent boys, which provides additional evidence for gender differences [62, 63].

In conclusion, this study documented the evidence of a relationship between the genotypes of *MTHFR* and childhood ALL risk and investigated age- and gender-interactions with the genotype to determine childhood ALL susceptibility. The presence of the T allele of C677T was not only a detectable and predictive biomarker for childhood ALL but also a protective determinant for older patients and boys.

## References

[1] Kaatsch P (2010) Epidemiology of childhood cancer. Cancer Treat Rev 36: 277–285. 10.1016/j.ctrv.2010.02.003 20231056

[2] Barry EV, Silverman LB (2008) Acute lymphoblastic leukemia in adolescents and young adults. Curr Hematol Malig Rep 3: 161–166. 10.1007/s11899-008-0023-9 20425461

[3] Belson M, Kingsley B, Holmes A (2007) Risk factors for acute leukemia in children: a review. Environ Health Perspect 115: 138–145. 17366834 10.1289/ehp.9023 PMC1817663

[4] Zwaan CM, Reinhardt D, Hitzler J, Vyas P (2010) Acute leukemias in children with Down syndrome. Hematol Oncol Clin North Am 24: 19–34. 10.1016/j.hoc.2009.11.009 20113894

[5] Mathew CG (2006) Fanconi anaemia genes and susceptibility to cancer. Oncogene 25: 5875–5884. 16998502 10.1038/sj.onc.1209878

[6] Szczepanski T, Harrison CJ, van Dongen JJ (2010) Genetic aberrations in paediatric acute leukaemias and implications for management of patients. Lancet Oncol 11: 880–889. 10.1016/S1470-2045(09)70369-9 20435517

[7] Bailey LB, Gregory JR (1999) Polymorphisms of methylenetetrahydrofolate reductase and other enzymes: metabolic significance, risks and impact on folate requirement. J Nutr 129: 919–922. 10222379 10.1093/jn/129.5.919

[8] Knock E, Deng L, Wu Q, Leclerc D, Wang XL, Rozen R (2006) Low dietary folate initiates intestinal tumors in mice, with altered expression of G2-M checkpoint regulators polo-like kinase 1 and cell division cycle 25c. Cancer Res 66: 10349–10356. 17079455 10.1158/0008-5472.CAN-06-2477

[9] Van der Put NM, Gabreels F, Stevens EM, Smeitink JA, Trijbels FJ, Eskes TK, et al (1998) A second common mutation in the methylenetetrahydrofolate reductase gene: an additional risk factor for neural-tube defects? AM J Hum Genet 62: 1044–1051. 9545395 10.1086/301825 PMC1377082

[10] Robien K, Ulrich CM (2003) 5,10-Methylenetetrahydrofolate reductase polymorphisms and leukemia risk: a HuGE minireview. Am J Epidemiol 157: 571–582. 12672676 10.1093/aje/kwg024

[11] Kang SS, Zhou J, Wong PW, Kowalisyn J, Strokosch G. (1988) Intermediate homocysteinemia: a thermolabile variant of methylenetetrahydrofolate reductase. AM J Hum Genet 43: 414–421. 3177384 PMC1715503

[12] Homberger A, Linnebank M, Winter C, Willenbring H, Marquardt T, Harms E, et al (2000) Genomic structure and transcript variants of the human methylenetetrahydrofolate reductase gene. Eur J Hum Genet 8: 725–729. 10980581 10.1038/sj.ejhg.5200522

[13] Sohn KJ, Croxford R, Yates Z, Lucock M, Kim YI (2004) Effect of the methylenetetrahydrofolate reductase C677T polymorphism on chemosensitivity of colon and breast cancer cells to 5-fluorouracil and methotrexate. J Natl Cancer Inst 96: 134–144. 14734703 10.1093/jnci/djh015

[14] Teng Z, Wang L, Cai S, Yu P, Wang J, Gong J, et al (2013) The 677C>T (rs1801133) polymorphism in the MTHFR gene contributes to colorectal cancer risk: a meta-analysis based on 71 research studies. PLoS One 8: e55332 10.1371/journal.pone.0055332 23437053 PMC3577825

[15] Yu L, Chen J (2012) Association of MTHFR Ala222Val (rs1801133) polymorphism and breast cancer susceptibility: An update meta-analysis based on 51 research studies. Diagn Pathol 7: 171 10.1186/1746-1596-7-171 23217001 PMC3536596

[16] Jia J, Ma Z, Wu S (2011) Positive association between MTHFR C677T polymorphism and oral cancer risk: a meta-analysis. Tumour Biol 35: 4943–4948. 10.1007/s13277-014-1650-5 24488626

[17] Tsai CW, Hsu CF, Tsai MH, Tsou YA, Hua CH, Chang WS, et al (2011) Methylenetetrahydrofolate reductase (MTHFR) genotype, smoking habit, metastasis and oral cancer in Taiwan. Anticancer Res 31: 2395–2399. 21737671

[18] Liu ZB, Wang LP, Shu J, Jin C, Lou ZX (2013) Methylenetetrahydrofolate reductase 677TT genotype might be associated with an increased lung cancer risk in Asians. Gene 515: 214–219. 10.1016/j.gene.2012.11.036 23237779

[19] Skibola CF, Smith MT, Kane E, Roman E, Rollinson S, Cartwright RA, et al (1999) Polymorphisms in the methylenetetrahydrofolate reductase gene are associated with susceptibility to acute leukemia in adults. Proc Natl Acad Sci U S A 96: 12810–12815. 10536004 10.1073/pnas.96.22.12810 PMC23109

[20] Matsuo K, Suzuki R, Hamajima N, Ogura M, Kagami Y, Taji H, et al (2001) Association between polymorphisms of folate- and methionine-metabolizing enzymes and susceptibility to malignant lymphoma. Blood 97: 3205–3209. 11342450 10.1182/blood.v97.10.3205

[21] Franco RF, Simoes BP, Tone LG, Gabellini SM, Zago MA, Falcao RP (2001) The methylenetetrahydrofolate reductase C677T gene polymorphism decreases the risk of childhood acute lymphocytic leukaemia. Br J Haematol 115: 616–618. 11736945 10.1046/j.1365-2141.2001.03140.x

[22] Wiemels JL, Smith RN, Taylor GM, Eden OB, Alexander FE, Greaves MF (2001) Methylenetetrahydrofolate reductase (MTHFR) polymorphisms and risk of molecularly defined subtypes of childhood acute leukemia. Proc Natl Acad Sci U S A 98: 4004–4009. 11274424 10.1073/pnas.061408298 PMC31169

[23] Wu KH, Wang CH, Yang YL, Peng CT, Lin WD, Tsai FJ (2010) Significant association of XRCC4 single nucleotide polymorphisms with childhood leukemia in Taiwan. Anticancer Res 30: 529–533. 20332465

[24] Bau DT, Tsai CW, Lin CC, Tsai RY, Tsai MH (2011) Association of *Alpha B-Crystallin* genotypes with oral cancer susceptibility, survival, and recurrence in Taiwan. PLoS One 6: e16374 10.1371/journal.pone.0016374 21915251 PMC3168435

[25] Wu HC, Chang CH, Tsai RY, Lin CH, Wang RF, Tsai CW, et al (2010) Significant association of methylenetetrahydrofolate reductase single nucleotide polymorphisms with prostate cancer susceptibility in Taiwan. Anticancer Res 30: 3573–3577. 20944139

[26] Kang S, Kim JW, Kang GH, Park NH, Song YS, Kang SB, et al (2005) Polymorphism in folate- and methionine-metabolizing enzyme and aberrant CpG island hypermethylation in uterine cervical cancer. Gynecol Oncol 96: 173–180. 15589597 10.1016/j.ygyno.2004.09.031

[27] Liu CS, Tsai CW, Hsia TC, Wang RF, Liu CJ, Hang LW, et al (2009) Interaction of methylenetetrahydrofolate reductase genotype and smoking habit in Taiwanese lung cancer patients. Cancer Genomics Proteomics 6: 325–329. 20065319

[28] Milne E, Royle JA, Miller M, Bower C, de Klerk NH, Bailey HD, et al (2010) Maternal folate and other vitamin supplementation during pregnancy and risk of acute lymphoblastic leukemia in the offspring. Int J Cancer 126: 2690–2699. 10.1002/ijc.24969 19839053

[29] Chan JY, Ugrasena DG, Lum DW, Lu Y, Yeoh AE (2011) Xenobiotic and folate pathway gene polymorphisms and risk of childhood acute lymphoblastic leukaemia in Javanese children. Hematol Oncol 29: 116–123. 10.1002/hon.965 20824655

[30] Damnjanovic T, Milicevic R, Novkovic T, Jovicic O, Bunjevacki V, Jekic B, et al (2010) Association between the methylenetetrahydrofolate reductase polymorphisms and risk of acute lymphoblastic leukemia in Serbian children. J Pediatr Hematol Oncol 32: e148–150. 10.1097/MPH.0b013e3181cbd252 20445408

[31] Tong N, Fang Y, Li J, Wang M, Lu Q, Wang S, et al (2010) Methylenetetrahydrofolate reductase polymorphisms, serum methylenetetrahydrofolate reductase levels, and risk of childhood acute lymphoblastic leukemia in a Chinese population. Cancer Sci 101: 782–786. 10.1111/j.1349-7006.2009.01429.x 20002681 PMC11159816

[32] de Jonge R, Tissing WJ, Hooijberg JH, Jansen G, Kaspers GJ, Lindemans J, et al (2009) Polymorphisms in folate-related genes and risk of pediatric acute lymphoblastic leukemia. Blood 113: 2284–2289. 10.1182/blood-2008-07-165928 19020309

[33] Kamel AM, Moussa HS, Ebid GT, Bu RR, Bhatia KG (2007) Synergistic effect of methyltetrahydrofolate reductase (MTHFR) C677T and A1298C polymorphism as risk modifiers of pediatric acute lymphoblastic leukemia. J Egypt Natl Canc Inst 19: 96–105. 19034339

[34] Chatzidakis K, Goulas A, Athanassiadou-Piperopoulou F, Fidani L, Koliouskas D, Mirtsou V (2006) Methylenetetrahydrofolate reductase C677T polymorphism: association with risk for childhood acute lymphoblastic leukemia and response during the initial phase of chemotherapy in greek patients. Pediatr Blood Cancer 47: 147–151. 16123993 10.1002/pbc.20574

[35] Zanrosso CW, Hatagima A, Emerenciano M, Ramos F, Figueiredo A, Felix TM, et al (2006) The role of methylenetetrahydrofolate reductase in acute lymphoblastic leukemia in a Brazilian mixed population. Leuk Res 30: 477–481. 10.1016/j.leukres.2009.01.019 16182363

[36] Krajinovic M, Lamothe S, Labuda D, Lemieux-Blanchard E, Theoret Y, Moghrabi A, et al (2004) Role of MTHFR genetic polymorphisms in the susceptibility to childhood acute lymphoblastic leukemia. Blood 103: 252–257. 12958073 10.1182/blood-2003-06-1794

[37] Silva RM, Fontes AC, Silva KA, Sant'Ana TA, Ramos FJ, Marques-Salles Tde J, et al (2013) Polymorphisms involved in folate metabolism pathways and the risk of the development of childhood acute leukemia. Genet Test Mol Biomarkers 17: 147–152. 10.1089/gtmb.2012.0174 23336575

[38] Sood S, Das R, Trehan A, Ahluwalia J, Sachdeva MU, Varma N, et al (2010) Methylenetetrahydrofolate reductase gene polymorphisms: association with risk for pediatric acute lymphoblastic leukemia in north Indians. Leuk Lymphoma 51: 928–932. 10.3109/10428191003719023 20367562

[39] Reddy H, Jamil K (2006) Polymorphisms in the MTHFR gene and their possible association with susceptibility to childhood acute lymphocytic leukemia in an Indian population. Leuk Lymphoma 47: 1333–1339. 16923565 10.1080/10428190600562773

[40] Amigou A, Rudant J, Orsi L, Goujon-Bellec S, Leverger G, Baruchel A, et al (2012) Folic acid supplementation, MTHFR and MTRR polymorphisms, and the risk of childhood leukemia: the ESCALE study (SFCE). Cancer Causes Control 23: 1265–1277. 10.1007/s10552-012-0004-0 22706675

[41] Azhar MR, Rahimi Z, Vaisi-Raygani A, Akramipour R, Madani H, Rahimi Z, et al (2012) Lack of association between MTHFR C677T and A1298C polymorphisms and risk of childhood acute lymphoblastic leukemia in the Kurdish population from Western Iran. Genet Test Mol Biomarkers 16: 198–202. 10.1089/gtmb.2011.0041 22017305

[42] Nikbakht M, MalekZadeh K, Kumar Jha A, Askari M, Marwaha RK, Kaul D, et al (2012) Polymorphisms of MTHFR and MTR genes are not related to susceptibility to childhood ALL in North India. Exp Oncol 34: 43–48. 22453148

[43] Metayer C, Scelo G, Chokkalingam AP, Barcellos LF, Aldrich MC, Chang JS, et al (2011) Genetic variants in the folate pathway and risk of childhood acute lymphoblastic leukemia. Cancer Causes Control 22: 1243–1258. 10.1007/s10552-011-9795-7 21748308 PMC3744066

[44] Lightfoot TJ, Johnston WT, Painter D, Simpson J, Roman E, Skibola CF, et al (2010) Genetic variation in the folate metabolic pathway and risk of childhood leukemia. Blood 115: 3923–3929. 10.1182/blood-2009-10-249722 20101025 PMC2869556

[45] Sadananda Adiga MN, Chandy S, Ramachandra N, Appaji L, Aruna Kumari BS, Ramaswamy G, et al (2010) Methylenetetrahydrofolate reductase gene polymorphisms and risk of acute lymphoblastic leukemia in children. Indian J Cancer 47: 40–45. 10.4103/0019-509X.58858 20071789

[46] Yeoh AE, Lu Y, Chan JY, Chan YH, Ariffin H, Kham SK, et al (2010) Genetic susceptibility to childhood acute lymphoblastic leukemia shows protection in Malay boys: results from the Malaysia-Singapore ALL Study Group. Leuk Res 34: 276–283. 10.1016/j.leukres.2009.07.003 19651439

[47] Alcasabas P, Ravindranath Y, Goyette G, Haller A, Del Rosario L, Lesaca-Medina MY, et al (2008) 5,10-methylenetetrahydrofolate reductase (MTHFR) polymorphisms and the risk of acute lymphoblastic leukemia (ALL) in Filipino children. Pediatr Blood Cancer 51: 178–182. 10.1002/pbc.21511 18421714

[48] Petra BG, Janez J, Vita D (2007) Gene-gene interactions in the folate metabolic pathway influence the risk for acute lymphoblastic leukemia in children. Leuk Lymphoma 48: 786–792. 17454638 10.1080/10428190601187711

[49] Kim NK, Chong SY, Jang MJ, Hong SH, Kim HS, Cho EK, et al (2006) Association of the methylenetetrahydrofolate reductase polymorphism in Korean patients with childhood acute lymphoblastic leukemia. Anticancer Res 26: 2879–2881. 16886608

[50] Oliveira E, Alves S, Quental S, Ferreira F, Norton L, Costa V, et al (2005) The MTHFR C677T and A1298C polymorphisms and susceptibility to childhood acute lymphoblastic leukemia in Portugal. J Pediatr Hematol Oncol 27: 425–429. 16096524 10.1097/01.mph.0000177513.81465.94

[51] Schnakenberg E, Mehles A, Cario G, Rehe K, Seidemann K, Schlegelberger B, et al (2005) Polymorphisms of methylenetetrahydrofolate reductase (MTHFR) and susceptibility to pediatric acute lymphoblastic leukemia in a German study population. BMC Med Genet 6: 23 15921520 10.1186/1471-2350-6-23 PMC1164414

[52] Thirumaran RK, Gast A, Flohr T, Burwinkel B, Bartram C, Hemminki K, et al (2005) MTHFR genetic polymorphisms and susceptibility to childhood acute lymphoblastic leukemia. Blood 106: 2590–2591; author reply 2591–2592. 16172254 10.1182/blood-2005-04-1719

[53] Balta G, Yuksek N, Ozyurek E, Ertem U, Hicsonmez G, Altay C, et al (2003) Characterization of MTHFR, GSTM1, GSTT1, GSTP1, and CYP1A1 genotypes in childhood acute leukemia. Am J Hematol 73: 154–160. 12827651 10.1002/ajh.10339

[54] Li X, Liao Q, Zhang S, Chen M (2014) Association of methylenetetrahytrofolate reductase (MTHFR) C677T and A1298C polymorphisms with the susceptibility of childhood acute lymphoblastic leukaemia (ALL) in Chinese population. Eur J Med Res 19: 5 10.1186/2047-783X-19-5 24476575 PMC3932997

[55] Yan J, Yin M, Dreyer ZE, Scheurer ME, Kamdar K, Wei Q, et al (2012) A meta-analysis of MTHFR C677T and A1298C polymorphisms and risk of acute lymphoblastic leukemia in children. Pediatr Blood Cancer 58: 513–518. 10.1002/pbc.23137 21495160

[56] Jiang Y, Hou J, Zhang Q, Jia ST, Wang BY, Zhang JH, et al (2013) The MTHFR C677T polymorphism and risk of acute lymphoblastic leukemia: an updated meta-analysis based on 37 case-control studies. Asian Pac J Cancer Prev 14: 6357–6362. 24377532 10.7314/apjcp.2013.14.11.6357

[57] Narayanan S, McConnell J, Little J, Sharp L, Piyathilake CJ, Powers H, et al (2004) Associations between two common variants C677T and A1298C in the methylenetetrahydrofolate reductase gene and measures of folate metabolism and DNA stability (strand breaks, misincorporated uracil, and DNA methylation status) in human lymphocytes in vivo. Cancer Epidemiol Biomarkers Prev 13: 1436–1443. 15342443

[58] Pereira AC, Schettert IT, Morandini Filho AA, Guerra-Shinohara EM, Krieger JE (2004) Methylenetetrahydrofolate reductase (MTHFR) c677t gene variant modulates the homocysteine folate correlation in a mild folate-deficient population. Clin Chim Acta 340: 99–105. 14734201 10.1016/j.cccn.2003.09.016

[59] Cartwright RA, Gurney KA, Moorman AV (2002) Sex ratios and the risks of haematological malignancies. Br J Haematol 118: 1071–1077. 12199787 10.1046/j.1365-2141.2002.03750.x

[60] Pearce MS, Parker L (2001) Childhood cancer registrations in the developing world: still more boys than girls. Int J Cancer 91: 402–406. 11169966 10.1002/1097-0215(200002)9999:9999<::aid-ijc1048>3.0.co;2-f

[61] Mossuz P, Cousin F, Castinel A, Chauvet M, Sotto MF, Polack B, et al (1998) Effects of two sex steroids (17beta estradiol and testosterone) on proliferation and clonal growth of the human monoblastic leukemia cell line, U937. Leuk Res 22: 1063–1072. 9783810 10.1016/s0145-2126(98)00101-5

[62] Papoutsakis C, Yiannakouris N, Manios Y, Papaconstantinou E, Magkos F, Schulpis KH, et al (2006) The effect of MTHFR(C677T) genotype on plasma homocysteine concentrations in healthy children is influenced by gender. Eur J Clin Nutr 60: 155–162. 16234842 10.1038/sj.ejcn.1602280

[63] Dierkes J, Jeckel A, Ambrosch A, Westphal S, Luley C, Boeing H (2001) Factors explaining the difference of total homocysteine between men and women in the European Investigation Into Cancer and Nutrition Potsdam study. Metabolism 50: 640–645. 11398138 10.1053/meta.2001.23286

